# The Impact of Helium and Nitrogen Plasmas on Electrospun Gelatin Nanofiber Scaffolds for Skin Tissue Engineering Applications

**DOI:** 10.3390/jfb15110326

**Published:** 2024-11-01

**Authors:** Abolfazl Mozaffari, Mazeyar Parvinzadeh Gashti, Farbod Alimohammadi, Mohammad Pousti

**Affiliations:** 1 Department of Textile Engineering, Yazd Branch, Islamic Azad University, Yazd 14515-775, Iran; 2 Department of Chemistry, Pittsburg State University, Pittsburg, KS 66762, USA; 3 National Institute for Materials Advancement, Pittsburg State University, Pittsburg, KS 66762, USA; 4 Department of Civil and Environmental Engineering, Temple University, Philadelphia, PA 19122, USA; farbod.alimohammadi@temple.edu; 5 Département de Chimie, Université Laval, 1045 Avenue de la Médecine, Québec, QC G1V 0A6, Canada; poustimohammad@gmail.com

**Keywords:** electrospinning, nanofiber, gelatin, helium and nitrogen gases, plasma, cell culture, density functional theory, molecular dynamics

## Abstract

This study explores the fabrication of tannic acid-crosslinked gelatin nanofibers via electrospinning, followed by helium and nitrogen plasma treatment to enhance their biofunctionality, which was assessed using fibroblast cells. The nanofibers were characterized using scanning electron microscopy, atomic force microscopy, attenuated total reflection Fourier transform infrared spectroscopy, X-ray diffraction, and water contact angle measurements before and after treatment. Helium and nitrogen gas plasma were employed to modify the nanofiber surfaces. Results indicated that helium and nitrogen plasma treatment significantly increased the hydrophilicity and biofunctionality of the nanofibers by 5.1° ± 0.6 and 15.6° ± 2.2, respectively, making them more suitable for human skin fibroblast applications. To investigate the impact of plasma treatment on gelatin, we employed a computational model using density functional theory with the B3LYP/6-31+G(d) method. This model represented gelatin as an amino acid chain composed of glycine, hydroxyproline, and proline, interacting with plasma particles. Vibrational analysis of these systems was used to interpret the vibrational spectra of untreated and plasma-treated gelatin. To further correlate with experimental findings, molecular dynamics simulations were performed on a system of three interacting gelatin chains. These simulations explored changes in amino acid bonding. The computational results align with experimental observations. Comprehensive analyses confirmed that these treatments improved hydrophilicity and biofunctionality, supporting the use of plasma-treated gelatin nanofibers in skin tissue engineering applications. Gelatin’s natural biopolymer properties and the versatility of plasma surface modification techniques underscore its potential in regenerating cartilage, skin, circulatory tissues, and hamstrings.

## 1. Introduction

Natural nanofibers, especially those based on gelatin, represent a promising area in biomedical applications. Gelatin, a natural polymer derived from collagen [1], is renowned for its excellent biocompatibility and biodegradability. These characteristics make it an ideal candidate for tissue engineering and regenerative medicine. Tissue engineering uses gelatin nanofibers to regenerate cartilage, skin, vascular tissues, and hamstrings [2,3,4,5,6,7,8]. Gelatin-based nanofibers can mimic the extracellular matrix, promoting cell adhesion, proliferation, and differentiation, which are crucial for effective tissue regeneration [9,10]. Since it is biocompatible, biodegradable, and readily available, gelatin is widely studied in electrospinning [11,12].

Electrospun nanofibers represent a revolutionary advancement in biomedical engineering. Their ability to mimic natural tissue structures, combined with their customizable properties and broad application potential, positions them as a cornerstone technology in regenerative medicine and other biomedical fields. As research progresses, addressing challenges such as mechanical strength and scalability will further enhance their applicability in clinical settings [9,13,14,15,16]. Meanwhile, electrospinning is a flexible and cost-effective approach for producing nanofibrous structures from natural and synthetic materials, showing great potential for future research [1,2]. Electrospinning is a common nanofiber production method due to its simplicity and efficiency [9]. Electrospinning uses several materials to make polymeric nanofibers for various purposes. These kinds of electrospun nanofibers are ideal for biological applications, including catalysis and tissue engineering, due to their high porosity, surface area, and tiny pore size [3,4,5,6,7,8]. As research continues to evolve in this field, gelatin nanofibers are likely to play an increasingly vital role in developing innovative solutions for medical challenges [9,10,13,14,16,17].

Mild solvents like acetic acid/water and ethanol/formic acid/water can be used in electrospinning solutions [6,18,19,20]. Due to their water sensitivity, gelatin nanofibers must be crosslinked to improve their mechanical and morphological qualities while retaining their three-dimensional integrity [21]. Chemical and enzymatic crosslinking improve tissue engineering [21,22,23,24,25,26]. Integrating tannic acid into gelatin nanofibers gives them antioxidant and antibacterial properties without requiring harmful crosslinking procedures [26,27,28]. Gelatin–tannic acid electrospun nanofibers were produced.

Plasma treatment, a popular surface modification approach, uses several methods and sources [28,29,30,31]. Electrons, radicals, photons, and charged ions make up plasma [32,33]. Strong electric fields generate ionized gases. Plasma is low- or high-temperature depending on pressure [34,35]. Plasma generation is also affected by discharge chamber and gas medium geometry. Plasma–polymer contact processes depend on particle density and collision frequency. Changing these parameters controls etching, functionalization, and crosslinking quality and quantity [35]. Plasma treatments are chemical-free and eco-friendly, and minimize heat effects under ideal circumstances, preventing damage to sensitive nanofibrous structures [30,36,37,38,39,40]. Grafting, roughening, and functionalization increase surface wettability in plasma modification [35,41,42]. Each plasma-producing device has its unique technique for creating and preserving plasma. Corona, dielectric barrier discharge (DBD), and radiofrequency plasma generators are examples [31]. Corona discharge plasma generators ionize air or gas around a conductor with high voltage [31,39]. DBD plasma generators create plasma with high reactive density by applying alternating current over a dielectric barrier [31]. Radiofrequency plasma systems precisely regulate plasma characteristics by ionizing gasses with electromagnetic fields [39]. Their capacity to create several reactive species aids subsequent reactions and surface modifications [39,43,44,45,46]. Plasma surface modification can improve biomaterial qualities by uniformly modifying exterior and interior structures [32,43,47]. Introducing functional groups like carboxylic acid, amine, and hydroxyl onto nanofiber surfaces with plasmas like argon, air-pressed, oxygen, nitrogen, ammonia, carbon dioxide, and helium plasma increases hydrophilicity and adhesion [32,33,38,48].

The temperature, electrical, and chemical properties of these treatments significantly alter surface changes [41]. Inert gases like argon scratch and bombard nanofibers, whereas compressed air improves hydrophilicity and interacts with polymers via oxygen-containing functional groups. Surface alteration occurs through bond breakdown and reformation or improved oxygen interaction post-treatment [49,50,51,52,53,54].

Helium plasma, which alters surfaces through numerous mechanisms at low temperatures and with high ionization efficiency, is ideal for applications requiring minimal thermal effect [55,56,57,58,59]. Plasma types and gas flow rates interact to inactivate pathogens differently with argon and helium gases [46,58]. Energy-efficient and environmentally friendly activation of nanofibrous tissue engineering scaffold surfaces is possible using nitrogen-containing plasmas. Reactive nitrogen species, especially nitric oxide, can enhance bacterial inactivation, wound healing, and regenerative therapy through direct chemical surface modification and electrostatic interactions [59,60,61,62].

After surface plasma treatments, scaffolds may retain their structural integrity and become hydrophilic, which can promote biomedical cell adhesion [63]. For gene therapy, medication administration, and cardiovascular and bone disease therapies, non-thermal plasma excels at biological temperatures [46,64].

For our investigation, we employed gelatin, a natural biopolymer commonly used in biofunctional applications. Thus, electrospinning was preferred for gelatin nanofiber production. Gelatin morphology is often improved by crosslinking. Tannic acid was used as a safe, natural chemical. In the next stage, plasma modification would boost gelatin nanofiber scaffold functioning, potentially impacting cell development. Our previous investigation employed argon, argon–oxygen, and air-pressed plasma gasses [49,65]. Helium and nitrogen gasses offer potential for bacterial inactivation and regenerative therapy; thus, we studied them [62,66,67,68]. This study examines plasma treatment with helium and nitrogen gasses for polymer and surface modification, highlighting their benefits and effects. The goal is to improve nanofiber characteristics for biological applications. Density functional theory (DFT) is becoming essential to skin tissue engineering. DFT helps develop and optimize skin regeneration biomaterials by aiding the understanding of their atomic and molecular electronic structure and characteristics. This computational method helps researchers anticipate biomaterial–biosystem interactions and build scaffolds with the desired biocompatibility, degradability, and mechanical strength. 

DFT may also be used to study cell–material interactions, wound healing, and skin regeneration. Researchers can better understand tissue development and healing by modeling the molecules and atoms involved in these processes. This knowledge can be used to develop effective skin regeneration and repair biomaterials and therapies [69].

Molecular dynamics (MD) simulations are essential for researching biomolecule structure, dynamics, and function. The reliability of these simulations depends on time scales and force field precision. The AMBER force field is essential for protein MD simulations, as it models complex peptide interactions using a robust and reliable parameter set developed over decades. This force field properly depicts a system’s potential energy as a function of atomic locations, simulating protein dynamics, folding, and molecular interactions. AMBER captures bond lengths, angles, torsions, van der Waals interactions, and electrostatic forces to study protein behavior at the atomic level, revealing biological processes and aiding in material design and development [70].

## 2. Materials and Methods

Tannic acid, 66% acetic acid, and Porcine Gelatin (from porcine skin with code G1890, Type A, powder, gel strength ~300 g Bloom, suitable for electrophoresis and cell culture) were provided by Sigma Aldrich (MO, USA). As previously described [26,71], we electrospun gelatin using acetic acid as the solvent. Ten milliliters of acetic acid was used to create a solution containing 15% *w*/*v* gelatin and 5% *w*/*v* tannic acid. The solution was shaken for four hours at thirty degrees Celsius to ensure homogeneity. The electrospinning procedure was conducted at a voltage of 15 kV, with a feeding rate of 0.6 mL/h and a needle tip distance of 15 cm from the metallic plate collector. The electrospinning apparatus was provided by Fanavaran Nano-meghyas Co. (Tehran, Iran).

After the nanofibers were collected, they were subjected to a vacuum oven at 45 °C for 3 h to create a fully crosslinked scaffold. Using the PF-200 plasma DBD equipment (Nik Fanavaran Plasma Co., Tehran, Iran), the electrospun nanofiber scaffolds were treated for ninety seconds in a plasma chamber filled with hydrogen and nitrogen gas. The flow rate for pure helium gas was two liters per minute. The nozzle and the sample were 3 mm apart, and the AC voltage was set to 10 kV. Gelatin scaffolds were created using the same electrospinning solution and treated with plasma gasses to evaluate the efficacy of the plasma modification process on crosslinked gelatin for tissue engineering applications.

More precisely, Dulbecco’s Modified Eagle’s Medium (DMEM; Biosera, England) was used to cultivate basic human dermal fibroblast cells. It was supplemented with 10% fetal bovine serum (FBS; Gibco, Belgium), 100 μg/mL streptomycin, and 100 IU/mL penicillin.

The cells were derived from fibroblasts obtained from human skin (Royan Institute, Tehran, Iran). The cells were maintained in a 5% CO_2_ atmosphere at 37 °C. Subsequently, gelatin films measuring 10 mm by 10 mm were used to culture the cells (fourth passage) under a microscope slide cover glass measuring 22 mm by 22 mm. We used 6-well cell culture plates with a seeding density of 10,000 cells/cm^2^ of media for this purpose. Cells were cultured, allowed to grow for 24 h, and then used for imaging with a microscope. To calculate the average number of cells in each captured image, we used an image processing software called ImageJ bundled with 64-bit Java 8. The average cell counts for each sample were determined by utilizing three images obtained from different locations. The morphology of the crosslinked gelatin nanofibers was examined using a scanning electron microscope (LEO1455VP, Cambridge, England). To carry this out, samples were vacuum coated with a Au layer using a sputtering technique before being examined under the microscope. We employed a pumping system in conjunction with an MTM-20 coating thickness controller (Haarlem, The Netherlands) that had a 30 W sputtering output. The target-to-substrate distance was 50 mm, and the coating thickness was 10 mm. For every sample, the scanning electron microscope (SEM)’s working distance was set to 9 mm, and two magnifications—×5 K and ×10 K (K = 1000)—were employed.

An accelerating voltage of 25 kV was used to operate the SEM. We provided the mean values of the five measurements that we surveyed [18]. The surface topography and nanofiber roughness in contact mode of the scanning probe microscope (SPM) (Park Scientific Instrument, Auto Probe CP Model, Korea) were measured using atomic force microscopy (AFM). To conduct this, the root mean square (RMS), which is the key variable in characterizing roughness, was computed using Equation (1):

(1) $$RMS=\frac{\sum_{n-1}^{N}(Zn-Zm)2}{N-1}$$

where *Zm* is the mean height and *Zn* is the height measurement of pixel n (out of a total of N = 256 × 256 pixels). FTIR spectroscopy was used to evaluate the chemical characteristics of the nanofibers (Thermo Nicolet NEXUS 870 FTIR from Nicolet Instrument Corp., Madison, WI, USA). The spectrophotometer was configured for reflection mode using single-reflection ATR bonding, and measurements were taken at a resolution of 5 cm^−1^ across a range of 500–4000 cm^−1^. The scaffolds’ capacity for absorbing water was tested using a water contact angle (CA) method supported by video camera equipment (Perkin Elmer Spectrum RX-1, Waltham, MA, USA). Wide-angle X-ray diffractograms obtained at 40 mA using a Philips X’Pert Pro Multipurpose X-ray Diffractometer were used to analyze the X-ray diffraction (XRD) of gelatin nanofibers. At 40 kV (k = 0.1542 nm), Ni-filtered Cu Kα radiation was produced, and at a scan speed of 1° per minute, the measured angle varied between 4° and 70°. All DFT calculations were performed using ORCA 6.0 software [19], and MD simulations were carried out with HyperChem 8.0 [20]. Geometries of untreated and plasma-treated gelatin were optimized using the BFGS quasi-Newton algorithm in redundant internal coordinates [21,22,23]. The B3LYP/6-31+G(d) level of theory was employed for DFT calculations, combining Becke’s three-parameter exchange functional (B3) [24] with the Lee–Yang–Parr correlation functional (LYP) [25]. For MD simulations, the Amber99 force field was utilized [26]. All computations were conducted in a vacuum environment. Initially, all models were fully optimized without constraints, followed by vibrational (IR) spectrum calculations at the same DFT level for the optimized structures. The high-resolution crystal structure (PDB ID: 1CAG) [27] was employed as the initial geometry after removing non-standard residues and water molecules. This structure, determined through X-ray crystallography, revealed a collagen-like peptide adopting a triple-helical conformation, corroborating previous fiber diffraction studies.

**Figure 1:**
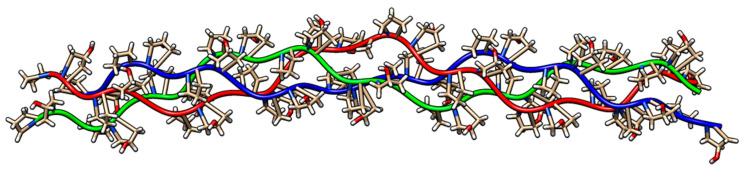
Optimized structure of a triple-helical collagen model composed of three 30-residue chains.

All-atom MD simulations, utilizing the AMBER force field (AMBERff) implemented in HyperChem, were conducted. Following initial geometry optimization with the Polak-Ribiere conjugate gradient algorithm (RMS gradient of 10^−3^ kcal/(Å mol)), the systems were heated to 300 K for 20 picoseconds and subsequently equilibrated for 100 nanoseconds. Figure 1 illustrates the optimized triple-helical collagen structure obtained using the AMBER force field.

**Figure 2:**
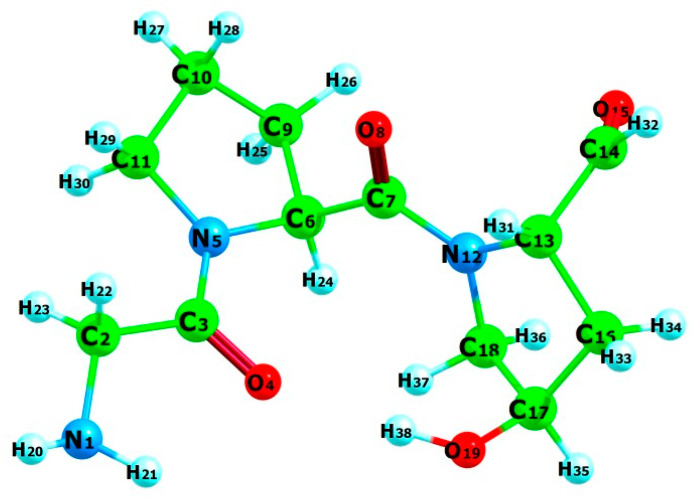
Optimized geometry of Gly-Pro-Hyp (GPH) at the B3LYP/6-31+G(d) level of theory. Color code: carbon (green), oxygen (red), nitrogen (blue), hydrogen (cyan).

Similarly to collagen, gelatin is primarily composed of repeating Gly-X-Y tripeptide units. Glycine constitutes approximately one-third of the amino acid sequence, while proline and hydroxyproline together make up another third [28]. To represent this structural motif, DFT calculations were performed on the Gly-Pro-Hyp (GPH) tripeptide. Figure 2 presents the optimized geometry of GPH, determined at the B3LYP/6-31+G(d) level of theory.

**Figure 3:**
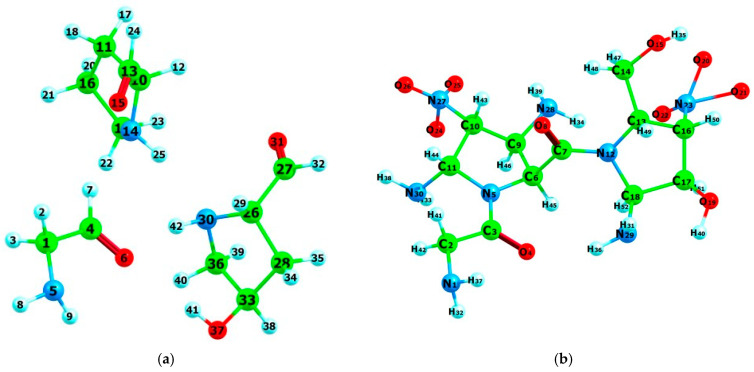
Optimized structure of plasma-treated GPH at B3LYP/6-31+G(d) with helium (**a**) and nitrogen (**b**). The occupied volumes are 293.98 Å^3^ and 378.24 Å^3^, respectively. Atoms are color-coded as follows: carbon (green), oxygen (red), nitrogen (blue), and hydrogen (cyan).

To investigate the impact of plasma treatment on gelatin, two models were proposed. Helium plasma treatment was simulated by selectively cleaving the C3-N5 and C7-N12 bonds within the gelatin chain. Nitrogen plasma treatment was modeled by randomly substituting hydrogen atoms with NH_2_ and NO_3_ groups [29]. The DFT-optimized structures of these modified gelatin models are depicted in Figure 3.

## 3. Results and Discussions

### 3.1. Microscopic Evaluation of Electrospun Gelatin Nanofibers

**Figure 4:**
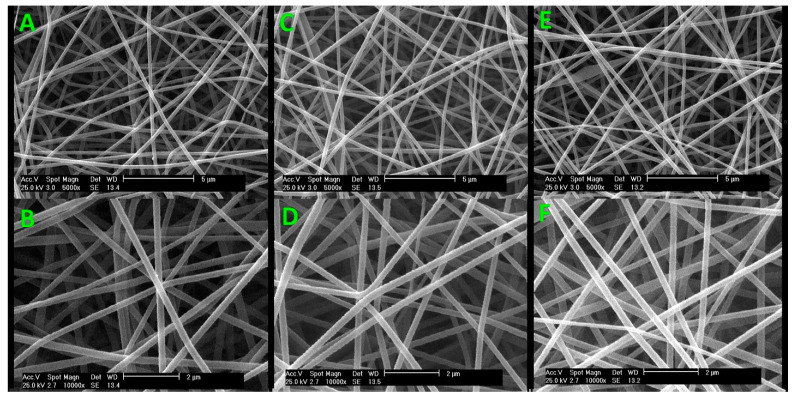
SEM images of electrospun gelatin nanofibers: plasma untreated at ×5000 (**A**), plasma untreated at ×10,000 (**B**), helium plasma treatment at ×5000 (**C**), helium plasma treatment at ×10,000 (**D**), nitrogen plasma treatment at ×5000 (**E**), and nitrogen plasma treatment at ×10,000 (**F**).

**Figure 5:**
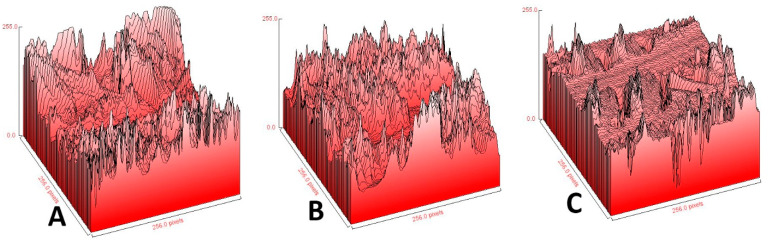
AFM image of untreated gelatin nanofibers (**A**), helium plasma-treated gelatin nanofibers (**B**), and nitrogen plasma-treated gelatin nanofibers (**C**).

The surface morphology of crosslinked gelatin nanofiber scaffolds was examined in their raw form, and after helium and nitrogen plasma processing, we used SEM and AFM techniques. Figure 4 displays SEM results for electrospun gelatin nanofibers before plasma treatment at ×5000 (A), before plasma treatment at ×10,000 (B), after helium plasma treatment at ×5000 (C), after helium plasma treatment at ×10,000 (D), after nitrogen plasma treatment at ×5000 (E), and after nitrogen plasma treatment at ×10,000 (F). Figure 5 shows the result of the AFM image of untreated gelatin nanofibers (A), helium plasma-treated gelatin nanofibers (B), and nitrogen plasma-treated gelatin nanofibers (C).

Our previous research on the production of tannic acid crosslinked gelatin nanofibers indicated that a balance of electrostatic repulsion, surface tension, and viscoelastic characteristics was essential for producing homogeneous nanofibers [30]. The median diameter of the nanofibers increases with higher concentrations of tannic acid in the gelatin bath [31]. There were no significant changes in the surface, shape, or mean diameter of nanofibers following plasma treatment [32]. Furthermore, the average diameter of gelatin/tannic acid nanofibers was found to be 100.6, highlighting the significance of this property in biomedical fields [33].

Thus, we adopted optimal electrospinning settings from our previous study to create smooth gelatin nanofibers [30,31]. In our most recent study on argon and argon–oxygen plasma-treated gelatin nanofibers, SEM showed no surface alterations, consistent with findings from air pressure plasma-treated nanofibers [30]. According to SEM observations, plasma treatment creates a mesh-like surface on biopolymer films [34]. The impact of solvent systems on the form of gelatin nanofibers has been extensively investigated elsewhere [35,36]. Similarly to previous research, the SEM images from this study showed no major changes in the surface morphology of the nanofibers following oxygen plasma treatment [37,38]. Notably, no significant alterations were observed in the nanofibers after modification by air plasma [39].

We investigated the surface characteristics of nanofibers using AFM, which generally provides reliable measurements of surface topography. We also analyzed AFM data, and the roughness parameters are displayed in Table 1. The surface of the untreated nanofibers was smooth, with an RMS of approximately 5.1 nm, as determined from Figure 4. The untreated sample (A), helium plasma-treated sample (B), and nitrogen plasma-treated sample (C) exhibited RMS measurements of 5.1 nm, 448.60 nm, and 40.31 nm, respectively.

One of the main causes of the immense rise in the roughness of nanofiber surfaces after plasma treatment is the bombardment of energetic particles such as electrons, ions, radicals, neutrals, and excited atoms/molecules. Chemical degradation, chain scission, and bond breaking result from the chemical etching of polymer surfaces, as shown by the plasma approach [49,52,71]. We discovered in several investigations that after plasma functionalization, the surface of polymer films and fibers can become oxidized [65]. Our previous examinations of other films and fibers yielded comparable results [30,32].

### 3.2. Chemical Analysis of Electrospun Gelatin Nanofibers

**Figure 6:**
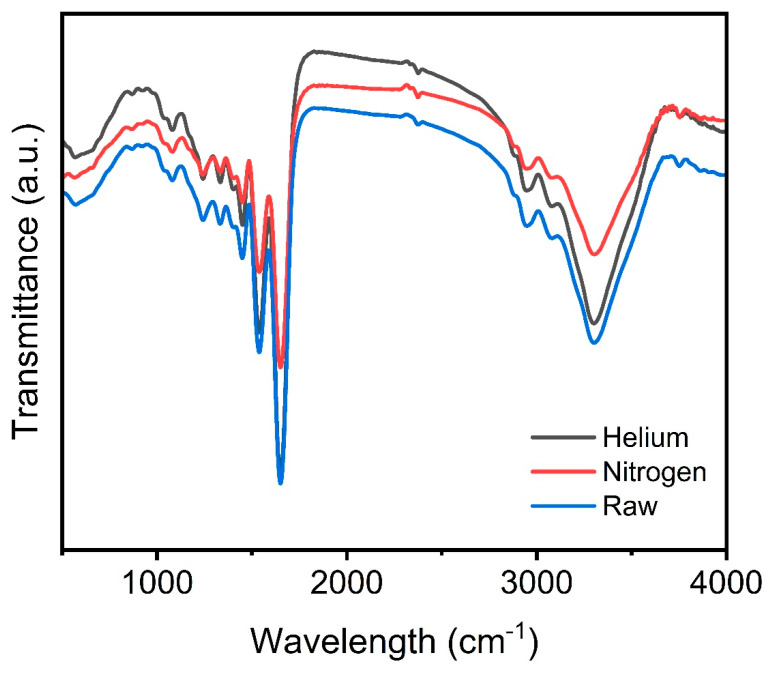
ATR-FTIR waveforms of untreated gelatin nanofibers, helium plasma-treated gelatin nanofibers, and nitrogen plasma-treated gelatin nanofibers.

The attenuated total reflection Fourier transform infrared (ATR-FTIR) spectra of crosslinked gelatin nanofibers, both prior to and following plasma gas processing, are shown in Figure 6. The primary peaks for the utilized samples are listed in Table 2. The FT-IR spectra of untreated gelatin nanofibers include peaks for aromatic C-H bending at 610 cm^−1^, C-H stretching at 2925 cm^−1^, and the N-H stretching of amide bonds at 3443 cm^−1^ [71,72,73,74,75].

Numerous investigations have detected the amide I band at 1650 cm^−1^, which is associated with the alpha–helix structures and unpredictable coils in protein-based materials [76,77,78,79,80]. Interestingly, the C-O stretching vibrations of peptide bonds, present in protein backbones, are linked to the amide I band [80,81,82,83,84,85]. The amide II band, at 610 cm^−1^, is associated with N-H out-of-plane wagging, C-N stretching vibrations, and N-H in-plane bending [86,87,88,89,90].

The amide II peak at 1538 cm^−1^ arises from N-H stretching in gelatin macromolecules [36,40,41]. The amide III band at 1334 cm^−1^ results from a combination of N-H in-plane bending, C-N stretching vibrations, and N-H out-of-plane wagging. C-O stretching bands are also present at 1242 and 1300 cm^−1^ [42,43,91,92,93,94,95].

A high-intensity band indicates that plasma treated with air as a gas mostly includes N2 species [44,96,97,98,99,100,101,102]. Therefore, it is more likely to be associated with the approximately 78% nitrogen component of air. The OH radical emission band can also be attributed to the ionization of water vapor in air. Meanwhile, the NO violet system shows a response between distinct reactive plasma molecules [44]. The prevalence of these reactive nitrogen species (RNS) is partly due to the Earth’s atmosphere being 78% nitrogen gas [44,45,46].

Gelatin nanofibers treated with helium plasma show C-H stretching at 3329 and 2948 cm^−1^. The overlap of N-H and O-H stretching vibrations results in a strong absorption band detected around 3300–3500 cm^−1^. At 1651 cm^−1^, C=O stretching vibrations (amide I) are found [84]. After being exposed to helium plasma, a band at 1540 cm^−1^, known as C=O stretching (amide I) and N-H bending vibrations (amide II), alters in amplitude. Following enhancement of gelatin nanofibers with helium plasma, the intensity of bands at 1449 (C-C bond), 1240, and 1076 cm^−1^ (C-O stretching bond) decreases again [65].

Gelatin nanofibers exposed to nitrogen plasma exhibit C-H stretching at 3303 and 2945 cm^−1^. A substantial absorption band is created around 3300–3500 cm^−1^ due to the overlap of N-H and O-H stretching vibrations. At 1651 cm^−1^, stretching vibrations of C=O (amide I) are detected. The band at 1538 cm^−1^ represents C=O stretching (amide I) and N-H bending vibrations (amide II). After being treated with nitrogen plasma, this band’s strength changes. Gelatin nanofibers functionalized with nitrogen plasma exhibit reduced band intensity at 1449 (C-C bond), 1241, and 1080 cm^−1^ (C-O stretching bond).

Table 2 depicts the effects of helium and nitrogen plasma functionalization on the primary gelatin bands.

Plasma can utilize a range of gasses, including argon, nitrogen, helium, and argon–oxygen mixtures, to create functional compounds or free radicals on material surfaces [47]. Nitrogen plasma treatment results in identical peaks to air plasma, except for a more prominent N-H bending band at 1250 cm^−1^ [39]. This is ascribed to the 3.3% nitrogen incorporated into the scaffolds’ surface as C-N and N-C=O bonds, as shown by XPS studies [48].

Nitrogen was discovered on the surface of PLA nanofibers electrospun from Atmospheric Pressure Plasma Jet-modified fluids containing nitrogen plasma [49]. However, several studies reported that oxygen was also implanted on the substrate’s surface following nitrogen plasma treatment [48,49,50]. Disparities in the literature regarding the location of similar nitrogen-bonded component peaks within the N1s envelope may be related to differences in binding energies [51]. Prior research was conducted to assess how nitrogen plasma affected the surface properties and cellular interactions of electrospun scaffolds [52,53,54,55].

### 3.3. Molecular Modeling of Electrospun Gelatin Nanofibers

**Figure 7:**
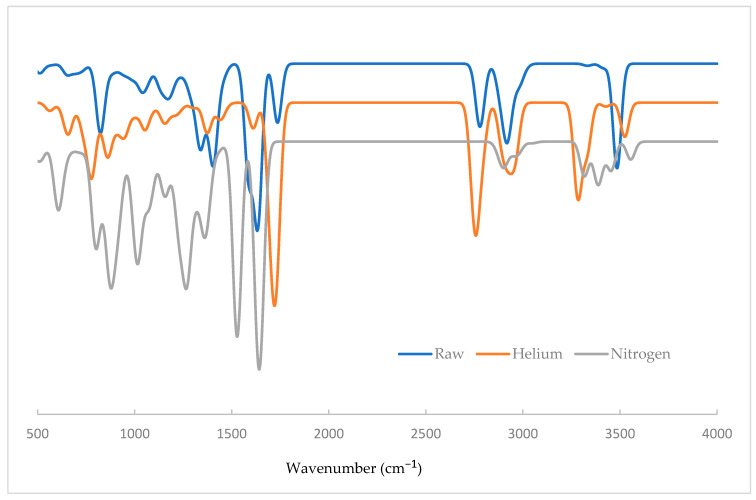
Calculated IR spectra of raw, helium plasma-, and nitrogen plasma-treated samples at B3LYP/6-31+G(d).

Figure 7 presents the calculated IR spectra of raw and (helium and nitrogen) plasma-treated gelatin. These spectra were generated using a full width at half maximum (FWHM) of 50 cm^−1^ and a scale factor of 0.96. The observable IR peaks predicted by the DFT model are provided in Table 3. The IR spectral data obtained from the proposed models demonstrate good agreement with the experimental results.

**Figure 8:**
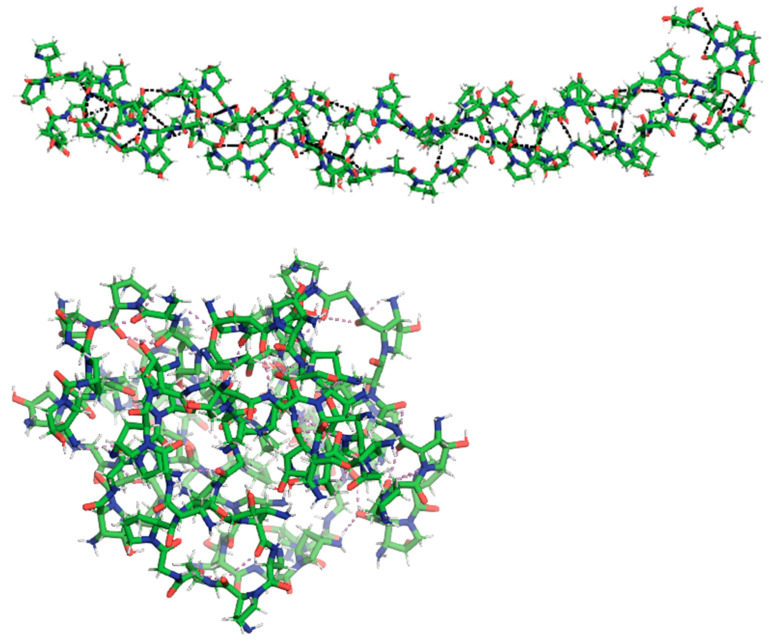
Comparison of optimized geometries for untreated (**top**) and nitrogen-treated (**bottom**) gelatin using the Amber99 force field. Atoms are color-coded: carbon (green), oxygen (red), nitrogen (blue), and hydrogen (white). Hydrogen bonds (polar contacts) are indicated by dotted lines.

To mimic the effects of nitrogen plasma treatment, eighteen amine (-NH_2_) groups were randomly incorporated into each of the three gelatin chains, totaling fifty-four additional amine groups. MD simulations revealed structural distortion in the plasma-treated gelatin compared to the preserved triple-helix conformation in the untreated gelatin. Analysis of hydrogen bonding demonstrated a 138% increase in polar contacts for the plasma-treated sample. Figure 8 illustrates the final geometries of both gelatin types after one hundred nanoseconds of simulation. The average number of hydrogen bonds (polar contacts), calculated using a 3.3 Å hydrogen–acceptor distance cutoff to encompass weaker interactions, was determined to be 54 for untreated gelatin and 129 for the plasma-treated variant during the final 2 nanoseconds of the simulation. The increased number of polar contacts in the plasma-treated gelatin indicates enhanced surface hydrophilicity, consistent with experimental findings.

### 3.4. X-Ray Diffraction Analysis of Electrospun Gelatin Nanofibers

**Figure 9:**
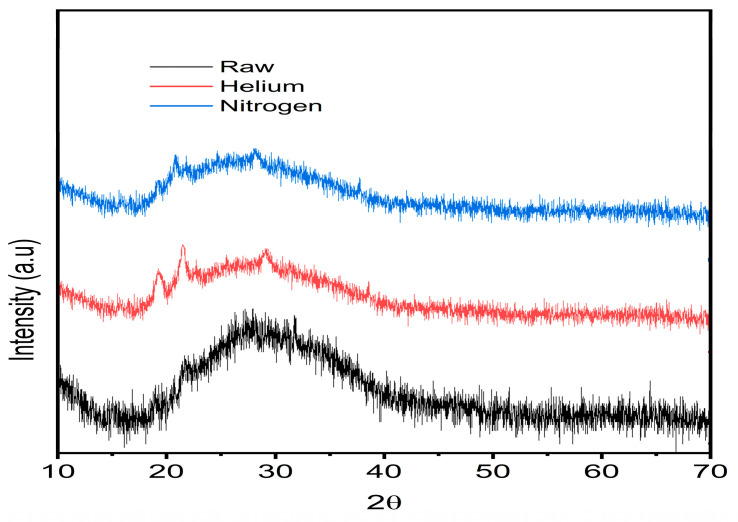
XRD spectra of untreated, helium plasma-treated, and nitrogen plasma-treated electrospun gelatin nanofibers.

The XRD experiment was conducted on untreated, helium plasma-treated, and nitrogen plasma-treated gelatin nanofibers, with the results displayed in Figure 9. Gelatin, being a partially crystalline biopolymer, typically shows a small peak at 2θ = 8° (d101 = 11.08 Å) and a broad peak at 2θ = 24° (d101 = 4.01 Å), which are associated with the triple-helical crystalline structure of gelatin biomacromolecules. Peña et al. identified that the crystalline structure of gelatin primarily arises from the triple-helix orientation of protein macromolecules [103]. In our experiment, the gelatin spectrum exhibited peaks at 2θ = 24°, 28.5°, 48°, and 65.5°, indicating that the three polypeptide chains of gelatin are stabilized by hydrogen bonds between the amino acids within each chain. However, the intense peak at 2θ = 8° was absent due to the addition of tannic acid as a crosslinker in the nanofiber processing (Figure 9 for Raw sample), as previously noted by Peña et al. [103].

After helium plasma treatment, three distinct peaks emerged at 2θ = 20°, 22°, and 30°, indicating a new biomacromolecular alignment on the nanofiber surface. This is due to the high-energy particles within the helium plasma breaking bonds on the surface of the gelatin nanofibers, creating reactive sites that can rearrange or form new bonds, leading to a change in the surface molecular orientation. In line with our findings, two research groups recently reported the appearance of new minor peaks in the XRD spectra of polyamide fibers following plasma activation [53,104].

Nitrogen plasma treatment resulted in similar but less intense peaks at 2θ = 20°, 22°, and 30°, suggesting a minor biomacromolecular orientation on the gelatin nanofiber surface compared to that of helium plasma.

The treatment of PLGA nanofibers with either oxygen plasma or ammonia plasma significantly lowered hydrophobicity and increased the number of polar groups on the nanofiber surface, as assessed using XPS analyses [56]. Following oxygen plasma treatment, there was a significant rise in the atomic proportion of oxygen bonded singly to carbon (C-O), likely due to the formation of hydroxyl or peroxyl groups on the PLGA nanofibers’ surface. It is also noteworthy that after treatment with ammonia plasma, the quantity of nitrogen atoms in PLGA nanofibers increased by up to 3.2% [56]. No noticeable changes in the crystallization of the nanofibers were observed after oxygen plasma treatment, highlighting the advantages of using low-temperature plasma for surface modification, as this process alters surface chemistry while preserving the polymer’s underlying physical structure [38,105,106,107,108,109,110].

### 3.5. Water Contact Angle Properties of Electrospun Gelatin Nanofibers

One of the most important properties of biomedical scaffolds is their hydrophilic or hydrophobic functionality [57]. The CA of the nanofiber scaffolds was measured using droplets of 0.5 mL, with three samples tested for each measurement. The mean value was reported. Gelatin, known for its hydrophilic properties, exhibited better wettability than other polymers such as polylactic acid and polycaprolactone, with a water contact angle of 20.65° due to its hydrophilic nature [58,110,111,112,113,114,115]. Table 4 shows the CA for untreated, helium plasma-treated, and nitrogen plasma-treated gelatin nanofibers. As expected, helium and nitrogen plasma treatments reduced the mean CA value to 5.1 ± 0.6° and 15.6° ± 2.2°, respectively, likely due to changes in nanofiber surface characteristics. The decrease in water contact angle indicates increased hydrophilicity, enhanced by the addition of -OH groups to the surface during plasma treatment [59]. Helium plasma treatment resulted in extremely hydrophilic gelatin nanofibers; water droplets were completely absorbed into the scaffold, as validated by our ATR-FTIR spectroscopy results. After plasma treatment, the surface becomes more wettable due to the grafting process, which enhances surface roughness or functionalization [60,61]. Nitrogen plasma treatment did not significantly alter surface topography or roughness values, yet it showed the most substantial change in wettability compared to helium, argon, and air plasmas [50]. The surface wettability of scaffolds transitioning from hydrophobic to hydrophilic states significantly improved the affinity of various cell types, such as Schwann cells and PC-12 cells [48,62,63]. The CA results demonstrated that nitrogen-containing gas plasma increased the surface hydrophilicity of PCL nanofibers less effectively than oxygen-containing gas plasma due to the lower dissociation energy of O_2_ (5.1 eV) compared to N_2_ (9.8 eV) [64,65]. The scaffolds formed by various polymeric fluids were subjected to DBD plasma in nitrogen at medium pressure [66,115,116,117,118,119,120]. Nitrogen plasma treatment significantly reduced the contact angle [67], and several experts acknowledged its use to enhance the nanofibers’ surface hydrophilicity [48,67]. After treatment in nitrogen-rich atmospheres (N_2_ and He/NH_3_), nanofibers exhibited hydrophilic surfaces with low water contact angles [68,69,70]. Increasing the plasma treatment time from 1 to 5 min significantly decreased the contact angle, indicating that the electrospun mats became more wettable. Generally, a polymer’s surface energy is higher when its wettability is greater. Oxygen plasma treatment enhances wettability by adding oxygenated polar functional groups (O, O_2_, O_2_−, COOH, C=O, etc.) to the surface of electrospun nanofibers [37,38,71,120,121,122,123,124,125].

Our latest study investigated the influence of gelatin crosslinking with tannic acid on CA characteristics. Tannic acid reduced the CA due to the crosslinking of functional groups at the scaffold surface and the presence of aromatic rings in the acid [31]. The most effective DBD processing strategy was determined by comparing the physicochemical and biological features of materials to improve the biocompatibility of PLA film matrices and human skin fibroblast cultures. The findings are applicable to cellular and tissue engineering technologies to promote skin and soft tissue regeneration [60]. Oxygen plasma-treated PCL scaffolds showed significant improvement in surface hydrophilicity, with water contact angle values decreasing from 130° to less than 20°. Argon plasma treatment of PCL scaffolds resulted in only a 20° drop in water contact angle values [62,72,73]. Martin and colleagues noted a significant decrease in water contact angle values following the treatment of electrospun polycaprolactone nanofibers [72]. By argon–oxygen plasma treatment, the surface of the nanofibers became hydrophilic [74,75]. Modifications to the surface chemical composition and roughness mostly contribute to increased material wettability [71,76,126,127,128,129,130].

In prior studies, after being treated with argon and oxygen plasma, PVA/Cs nanofibers showed a rapid decrease in contact angle, attributed to the high reactivity of oxygen ions, which ultimately causes high surface roughness and the increased incorporation of functional groups containing oxygen on the surface [71]. Oxygen plasma treatment considerably increases the wettability of nanofibers by reducing surface roughness and the water contact angle from 73° to 25°. However, this unequivocally shows that oxygenated functional groups produced during oxygen plasma treatment are incorporated onto the nanofibers’ surface, enabling water molecules to interact forcefully with these polar functional groups, thus decreasing the contact angle [38,77,78,79].

### 3.6. Biocompatibility of Treated Electrospun Gelatin Nanofiber Scaffolds

**Figure 10:**
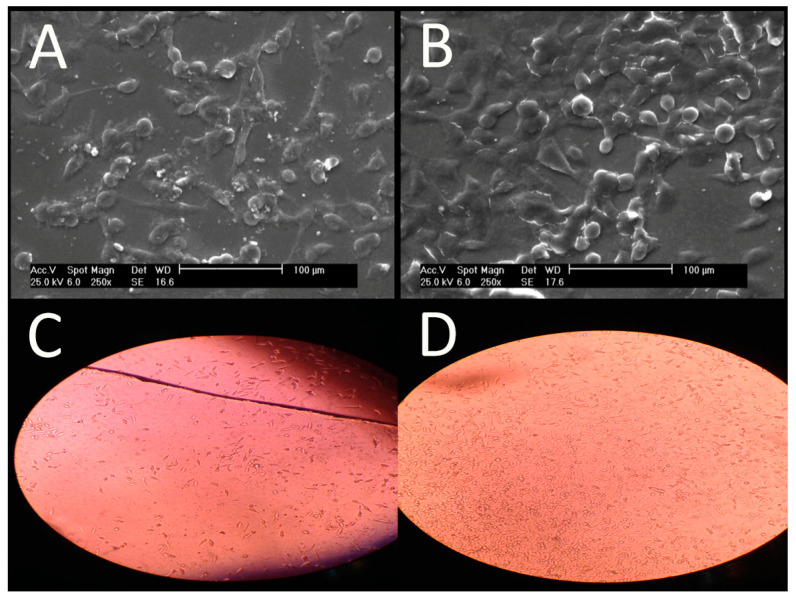
Images of fibroblast cells on a gelatin scaffold: (**A**) a SEM image of the helium plasma-treated gelatin scaffold; (**B**) a SEM image of the nitrogen plasma-treated gelatin scaffold; (**C**) an inverted optical microscope image of the helium plasma-treated gelatin scaffolds at 250× magnification; and (**D**) an inverted optical microscope image of the nitrogen plasma-treated gelatin scaffold at 250× magnification.

Over 24 h, fibroblast cells were cultured on scaffolds and observed using both a SEM and an inverted optical microscope. SEM imaging (Figure 10A) demonstrated that fibroblast cells adhered well to the helium plasma-treated gelatin scaffolds and maintained their normal morphology even after a 24 h of trypsin treatment. However, the cell shape remained unchanged on the nitrogen plasma-treated gelatin scaffold (Figure 10B). The inverted microscope images (Figure 10C,D, at 250× magnification) showed flat fibroblast cells on both helium and nitrogen plasma-treated scaffolds. Remarkably, plasma functionalization was found to increase the cell density on gelatin, a finding consistent with those of our latest research on argon, argon–oxygen, nitrogen, and air plasma-treated gelatin nanofibers [30,32,80]. The build-up of reactive oxygen and nitrogen species on the nanofiber surface caused materials and bacteria to interact electrostatically [81,82]. PC-12 cells were used to assess the impact of these beneficial plasma-induced modifications on the functionality of electro-sensitive cells [48]. Significantly enhanced cell viability, metabolic activity, adhesion, and proliferation were observed on nanofibers modified by helium plasma, which are critical for applications in nerve tissue regeneration, disease modeling, and drug testing [83]. Plasma surface modifications of tissue engineering scaffolds show great potential for improving cell–scaffold interactions [62]. Quantitative in vivo studies with fibroblast cells by Barry and colleagues demonstrated that plasma-modified polylactic acid had significantly higher cell adhesion capabilities compared to its unmodified counterpart [84,85]. Plasma alteration is crucial in enhancing the hydrophilicity of scaffold surfaces, promoting osteoblastic cell proliferation and differentiation [86,87,88]. Plasma treatment conditions resulted in the increased adhesion and proliferation of fibroblasts, chondrocytes, and osteoblasts compared to the untreated electrospun PCL scaffold [73]. Asadian et al. utilized argon plasma to modify the surface properties of electrospun PCL nanofibrous meshes, thereby controlling cell behavior [55]. Studies have also investigated how nitrogen plasma affects the surface properties and cellular interactions of electrospun nanofiber scaffolds [52,54,55,62,69]. Biocompatibility experiments showed improved adhesion and proliferation on collagen nanofibers treated by plasma [89]. The cell compatibility of samples whose surfaces were modified with a blend of argon and nitrogen plasma was assessed in vitro using the L929 fibroblast cell line. It was demonstrated that plasma treatment significantly influences the scaffolds’ initial cell attachment, crucial for long-term cell compatibility [90]. On ammonia plasma-treated nanofibers, fibroblast cell adhesion and proliferation were markedly increased [56]. After one week, it was found that argon and nitrogen plasma treatments significantly increased cell proliferation, while helium and dry air plasma treatments had a minor effect. Another observation was that nitrogen plasma significantly enhanced the treated surface’s bioactivity [50]. Oxygen plasma-treated nanofibers not only exhibited strong wettability but also demonstrated antibacterial efficacy against Gram-positive *S. aureus* and Gram-negative *E. coli* bacteria [37].

Argon and oxygen plasma surface treatment of PVA/Cs nanofibers has increased their mechanical strength and biocompatibility for use in biomedical applications. Culture results indicate that nanofibers treated with oxygen plasma have a higher degree of hydrophilicity than those treated with argon, potentially enhancing their cytocompatibility [71,130,131,132,133].

## 4. Conclusions

We investigated the influence of gelatin nanofibers treated with helium and nitrogen gas plasma on their physical, chemical, and biofunctional properties. Nanofiber surface roughness rose considerably following helium and nitrogen plasma treatment due to ionization and chemical degradation. Furthermore, ATR-FTIR spectroscopy was used to survey a variety of peaks. The GPH model was subjected to DFT calculations using the B3LYP functional and 6-31+G(d) basis set, both with and without simulated plasma treatment. Optimized geometries and IR spectra were determined for each system. Calculated IR frequencies closely matched experimental data for both raw and plasma-treated gelatin. MD simulations indicated an increase in polar contacts for plasma-treated gelatin, suggesting enhanced surface hydrophilicity, consistent with experimental observations. In contrast to our earlier investigation on argon and argon–oxygen plasma-treated gelatin nanofiber scaffolds, we found no significant alterations in the XRD patterns of gelatin nanofiber scaffolds after nitrogen plasma treatment. 

Gelatin’s water CA dropped significantly from 20.65° to 5.1° following helium plasma treatment. The nitrogen contact angle sank to 15.6°, similar to previous studies on argon, argon–oxygen, and air-pressed plasma treatments [30,32]. This could be related to the insertion of -OH groups on the surfaces of scaffolds. Notably, our technique improved hydrophilicity, as previously established. Finally, the number of fibroblast cells increased on gelatin treated with both plasma gasses [50]. We conclude that plasma functionalization is necessary for skin tissue regeneration.

## Figures and Tables

**Table 1:** RMS roughness values as an effect of helium and nitrogen plasma usage on electrospun gelatin nanofibers.

Samples	Duration of Treatment	R_a_ (nm)	R_z_ (nm)	R_q_ (nm)	R_max_ (nm)
Untreated	0 s	6.585	276.8	47.48	5.1
Helium-treated	90 s	20.21	252.8	180.2	448.60
Nitrogen-treated	90 s	3.921	25.54	14.85	40.31

**Table 2:** ATR-FTIR peak assignments for gelatin nanofibers before and following helium and nitrogen plasmas treatment.

Gelatin Nanofibers		Helium Plasma Treatment of Gelatin Nanofibers		Nitrogen Plasma Treatment of Gelatin Nanofibers	
Peak Position (cm^−1^)	Band Assignment	Peak Position (cm^−1^)	Band Assignment	Peak Position (cm^−1^)	Band Assignment
610–669 1242	−CH bending C−O stretching	1076 1240	C−O stretching C−O stretching	1080 1241	C−O stretching C−O stretching
1300	C−O stretching	1449	C−C	1449	C−C
1334	C−N stretching/Amide II	1540	N−O stretching/Amide II	1333	C−N stretching/Amide III
				1538	N−O stretching/Amide II
1538	N−O stretching/Amide II	1651	C−O stretching/Amide II	1650	C−O stretching/Amide II
2925	−CH stretching	2948	−CH stretching	2945	−CH stretching
3443	O−H stretching/Amide A	3300–3500	O−H stretching/Amide A	3300–3500	O−H stretching/Amide A

**Table 3:** Calculated IR peak frequencies for raw and plasma-treated gelatin.

Peak Position (cm^−1^)			Band Assignment
Raw Gelatin	Helium-Treated Gelatin	Nitrogen-Treated Gelatin	
665	654		−CH bending
1338	1211	1265	C−N stretching/Amide III
1591		1529	N−O stretching/Amide II
1630	1609	1645	C−O stretching/Amide II
2918	2940	2900	−CH stretching
3482	3435	3456	O−H stretching/Amide A

**Table 4:** Water contact angle of untreated gelatin nanofiber scaffold (A), helium plasma-treated gelatin nanofiber scaffold (B), and nitrogen plasma-treated scaffolds (C).

	A	B	C
Average Water Contact Angle (°)	20.7 ± 6.1	5.1 ± 0.6	15.6 ± 2.2
Schematic Diagram	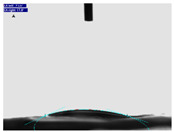	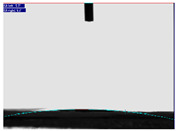	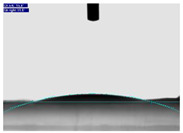

## Data Availability

The original contributions presented in the study are included in the article, further inquiries can be directed to the corresponding authors.
